# The sequoia-loving sprite, a new genus and species of fungus gnat (Diptera, Mycetophilidae) from California

**DOI:** 10.3897/zookeys.437.7932

**Published:** 2014-08-28

**Authors:** Peter H. Kerr

**Affiliations:** 1 California State Collection of Arthropods, California Department of Food and Agriculture, 3294 Meadowview Rd., Sacramento, CA, 95832–1448 USA

**Keywords:** Systematics, fungus gnats, new genus, new species, California Floristic Province, *Spritella*, giant sequoia tree, *Sequoiadendron giganteum*

## Abstract

California is one of the most biologically diverse regions of the world, yet the diversity of fungus gnats (Mycetophilidae) remains largely undocumented within the state. A modest survey of these flies has led to the discovery of a new genus and species of gnat that lives alongside one of the most iconic trees in the world, the giant sequoia (*Sequoiadendron giganteum*). *Spritella sequoiaphila* **gen. et sp. n.** is described and illustrated and its status among other mycetophilid genera is analyzed and discussed.

## Introduction

California is home to one of the most biologically diverse regions of the world (Myers et al. 2000), although it is unknown exactly how many species of Mycetophilidae (Diptera) occupy the state. Recent monographs suggest that the family remains in need of rigorous taxonomic development. For example, the genera *Novakia* Strobl and *Azana* Walker were not known to occur in the state until 2007 and 2010, respectively (representing four new species; Kerr 2007, 2010); all three species of *Acomoptera* Vockeroth living in California were undescribed until 2011 (Kerr 2011); both species of Californian *Phthinia* Winnertz were not described until 2014 (Fitzgerald and Kerr in press); and an additional seven new species (six of these California endemics) of *Megophthalmidia* Dziedzicki were only just recently discovered and described (Kerr 2014).

Our understanding of the diversity and distribution of Mycetophilidae and related flies in California will remain limited as long as material available for study is lacking in museums and other curated collections. In an effort to document latent diversity within the California Floristic Province, a modest collecting program has been conducted throughout the state over the last several years. Although woefully incomplete, this effort has generated over 5000 genus-level specimen identifications of fungus gnat specimens from over 170 different collecting events (nearly all Malaise traps) in 12 different California counties.

One of the most iconic symbols of the California Floristic Province is the giant sequoia groves of the Sierra Nevada Mountains. This habitat is special for its natural inhabitants as much as for the emotional reaction it inspires. The giant sequoia is the planet’s largest living organism and found only in California, with some trees towering over 300 feet tall and having trunk diameters of over 55 feet. In 1853, when the ‘Mother of the Forest’ tree was cut down in Calaveras County for speculative commercial exploitation, it set off the first national awakening of environmentalist sentiments that called for the protections of public lands. This awareness, fostered by the popular publications of naturalist John Muir, led to the first protections of these trees in 1864 (Mariposa Grove in Yosemite Valley), and eventually led to the establishment of the United States National Park Service in 1872. Today, the magnificent ‘Big Trees’ bring over 5,000,000 people per year to visit parks that contain giant sequoia groves, generating tens of millions of dollars in tourist revenue for local economies every day (Thomas et al. 2014).

It was within giant sequoia forest habitat that an especially curious fungus gnat was recently discovered. Because of its unusual morphology – particularly, the unique wing venation of the male – the phylogenetic affiliation of this fly was not immediately clear. This paper describes this species, illustrates its morphology, and locates it within a systematic phylogenetic framework among currently known Mycetophilidae.

## Materials and methods

Terminology for thoracic, wing, and genitalic morphology is consistent with Kerr 2011, which follows Söli (1997), McAlpine (1981), Vockeroth (1981), and Matile (1990). The terms “genitalia” and “terminalia” are used interchangeably. Genitalia were macerated in 10% KOH at approx. 95 °C for 15–20 minutes to remove soft tissue, then rinsed in distilled water and dilute glacial acetic acid, and dissected in water. All genitalia preparations were placed in a small genitalia vial containing glycerol, and pinned beneath the specimen. Figures were made using Adobe Illustrator and Adobe Photoshop Creative Suite software, with digital images taken using a Nikon DS-Fi1 scope-mounted digital camera. Habitus images were taken with the same digital camera (or the Nikon DS-Fi2), using an LED dome lighting system (Kerr et al. 2008). Material examined is deposited in the California State Collection of Arthropods, Sacramento, CA (CSCA), as indicated in square brackets after the transcribed specimen label data.

All measurements are made in millimeters. Ranges are given for body length, wing length, and the mean for each of these values is provided. Measurements of the holotype are given in square brackets. The number of individuals measured is noted in parentheses. All measurements are of critical-point dried specimens.

### Key to the sciophiline genera lacking a posterior wing vein fork (Mycetophilidae: Sciophilinae: “*Azana* group” *sensu* Matile 1998) (Modified from Matile 1998)

**Table:** 

1	Posterior vein forked, sometimes incomplete at the base; mediotergite setose or not	(Other Sciophilinae)
–	Only one posterior wing vein; mediotergite setose	2
2 (1)	M_2_ absent or present in the form of a vein more or less obsolete at the base	8
–	Anterior fork complete	3
3 (2)	Wing macrotrichia oriented toward base of wing; sc-r and R_4_ present (R_4_ occasionally absent); metepisternum with fine setae	*Monoclona* Mik
–	Wing macrotrichia oriented toward wing apex; sc-r and R_4_ present or absent; metepisternum bare	4
4 (3)	Sc-r present, R_4_ present or absent; tibia II without sensory organ	6
–	Sc-r and R_4_ absent; tibia II bearing sensory organ	5
5 (4)	Subcostal wing vein not exceeding (or just barely exceeding) the apex of the basal cell; second palpal segment strongly dilated; male tergite IX large, lacking modified setae	*Cluzobra* Edwards
–	Subcostal wing vein clearly exceeding the apex of the basal cell; second palpal segment weakly dilated; male tergite IX short, bearing a pair of modified paddle-shaped bristles	*Afrocnemia* Matile
6 (4)	R_4_ present; anterior basalare setose; anepisternum bearing short setae; subcostal vein usually less than half length of wing membrane	*Parvicellula* Marshall
–	R_4_ absent; anterior basalare bare; anepisternum bare or bearing short setae; subcostal vein usually half length of wing membrane or longer	7
7 (6)	Anepisternum bare or with small setae; katepisternum bare; sc-r proximal of Rs	*Acnemia* Winnertz
–	Anepisternum with small setae near upper margin; katepisternum with setae; sc-r distad of Rs	*Spritella* gen. n.
8 (2)	Subcostal very short, free at apex; r-m longitudinal, the basal cell small and rectangular	*Azana* Walker
–	Subcostal ending in C or R, r-m longitudinal or not	9
9 (8)	Subcostal long, ending in R; r-m long and longitudinal; anepisternum setose	*Neotrizygia* Tonnoir & Edwards
–	Subcostal short, ending in C; r-m long or short; anepisternum setose or bare	10
10 (9)	Sc-r present, anepisternum setose	*Trizygia* Skuse
–	Sc-r absent, anepisternum bare	11
11 (10)	Wing macrotrichia directed toward the wing apex; r-m oblique; M_1_ entire; R_4_ present (New Zealand) or absent (South America)	*Paratrizygia* Tonnoir
–	Wing macrotrichia directed toward the wing base; r-m longitudinal, the basal cell small rectangular; M_1_ incomplete at the base; R_4_ absent	*Neoaphelomera* Miller

## Taxonomy

### Spritella gen. n.

Taxon classificationAnimaliaDipteraMycetophilidae

http://zoobank.org/BAA00509-5AFE-42E3-9A05-B10FE54A83E3

#### Type species.

*Spritella sequoiaphila* gen. et sp. n., by current designation.

#### Diagnosis.

Three ocelli, antennae with 14 cylindrical flagellomeres, maxillary palpus 4-segmented, scutum raised above level of head, upper half of anepisternum with setae, ventro-posterior area of katepisternum with microsetae, tibial spurs 1:2:2. Wing membrane with macrotrichia; costa produced beyond tip of R_5_; subcosta long, ending at C, approximately at midpoint of wing; sc-r present, arising beyond origin of Rs; r-m missing because Rs and M_1_ touching or r-m present, short; M_2_ arising from discal cell basad of origin of Rs or at base of M_1_; cubital vein unforked, A_1_ well developed, reaching beyond origin of Rs. Male gonostylus without basal appendages.

*Spritella* gen. n. resembles *Acnemia* Winnertz by its lack of a posterior fork and foreshortened medial stem. However, the new genus is readily separated from *Acnemia* by the presence of setae on the anepisternum and katepisternum; sc-r arising well beyond origin of Rs; and by having male gonostylus without a basal process. Other sciophiline genera that also lack the cubital fork include *Afrocnemia* Matile, *Cluzobra* Edwards, *Monoclona* Mik, and *Parvicellula* Marshall. In the new genus, crossvein sc-r is clearly present unlike in *Afrocnemia* and *Cluzobra*; R_4_ is absent unlike in *Parvicellula*; and the macrotrichia of wing membrane are decumbent, directed toward wing apex unlike in *Monoclona*. The long subcostal vein of *Spritella* gen. n. (relative to wing length) and position of sc-r relative to Rs is also distinctively different from these genera.

#### Description.

**Head** shape in anterior view subequal, approximately as long as wide; medial eye margins farther apart dorsally than ventrally; antennal eye notch present, at least two ommatidia deep; interommatidial setulae present between all ommatidia; ocelli three, nearly linear; lateral ocellus between 1× and 1.5× its own diameter from eye margin, between 2.5× and 3× its own distance from median ocellus; all ocelli dorsad of eye margin; occipital suture from median ocellus to occiput absent; frontal suture between median ocellus and ventral margin of frons complete, suture between lateral ocelli and eyes also present; frons with setae; face approximately 2× longer than wide, parallel-sided along most of length, bearing setulae throughout; face and clypeus separated by complete suture; clypeus ovate, approximately one-half length of face, covered with short setae. Antennal scape and pedicel subequal in size; scape with setae approximately 2× scape length; pedicel setae approximate length of pedicel; antennal flagellomeres 14, cylindrical, approximately 3× longer than wide, approximately the same length but thinner distally, densely covered with short setae. Palp with 4 visible segments, none with apparent sensory pit.

**Thorax** (Fig. 2) raised, scutum dorsad of head position; short setae distributed throughout scutum, acrostichal setae present, bristles present along lateral margins of scutum; postalar wall and callus separated by carina; scutellum clearly wider than long, narrower than scutum; antepronotum and proepisternum with bristles; anepisternum with setae dorsally; anterior basalare bare; anapleural suture incomplete; katepisternum with setae ventro-posteriorly; anepimeron bare; anepisternum with few inconspicuous setulae; laterotergite raised ventrally, with bristles and shorter setae; metepisternum bare; mediotergite with three bands of bristles ventrally and shorter setae that extend along dorsoventral length, medially. **Wing** membrane covered with microtrichia and macrotrichia that are arranged irregularly; C ending beyond R_5_; dorsal surface of humeral vein without setae, ventral surface with setae; subcostal vein setose on both sides, ending in C, approximately at midpoint of wing; sc-r present, arising distad of origin of Rs; R_1_ setose on dorsal and ventral surfaces, although bare basad of Rs vein ventrally; vestigial M vein within discal cell present or absent; R_4_ not present, r-m present or absent (R_5_ joining M_1_ at junction with Rs); M_1_ setose above, bare below; M_2_ setose above, bare below, either arising from bM, from junction of M_1_ and Rs, or from base of M_1_; cubital vein unforked, setose above, bare below, ending at wing margin; CuP strong at base, extending apically as weak fold; anal vein strong, setose on both sides (less so ventrally), extending beyond origin of Rs. **Legs** elongate; coxae with dark, erect setae and lighter, shorter decumbent setae; femora with short, appressed setae and microtrichia; mid tibial organ absent; tibial spur formula 1:2:2; tibiae with short, appressed setulae and short, erect setae that are no longer than half widest width of tibia; tarsal claws small; empodium developed.

**Abdomen** with segments of subequal width; sternites with two longitudinal fold lines along length; in male, segments 8 modified so that genitalia orient upwards. **Male terminalia** with enlarged, hood-like epandrial sclerite (tergite IX); cerci and epiproct reduced; hypoproct with lightly sclerotized anterior apodemes; gonocoxites widely separated, joined by narrow medial bridge; gonostyli simple, without subtending appendages, inwardly-directed, and arising from middle area of gonocoxites. **Female terminalia** with first cerci elongate, second cerci ovoid, sternite 8 clearly larger than tergite 8.

#### Etymology.

The genus name is feminine, derived from the English “sprite” and the Latin ending “-ella”, as a diminutive.

### Spritella sequoiaphila sp. n.

Taxon classificationAnimaliaDipteraMycetophilidae

http://zoobank.org/8135BBEC-1424-401B-9320-53205C3AD187

Figs 1 [Fig F2] [Fig F3] [Fig F4] [Fig F5] [Fig F6] [Fig F7] [Fig F8] –13

#### Type material.

Holotype: ♂, “USA: CA: USA: CA: Tulare Co.: Whitaker Forest, EshomCrk.Drainage, nr. tree#142, 36.7062°N, -118.9319°W, 1650masl, YPT, 3.vi–16.vii.2010 P.H. Kerr” / “HOLOTYPE 10F761♂ *Spritella sequoiaphila* Kerr 2014” [red label]. Deposited in CSCA, dissected specimen mounted on gray point, terminalia in glass vial marked “10F761 HT” on pin below specimen. Type locality indicated in Fig. 14.

Paratypes: 1 ♂, USA: CA: Calaveras Co., Calaveras Big Trees SP, S. grove fire rd., nr. Beaver Creek, MT#1, 38°15.41'N, 120°15.25'W 1385masl, 22.v.–11.vi.2007 P.H. Kerr & A.R. Cline 07LOT086” [CSCA]; 7 ♂♂, 8 ♀, “USA: CA: Tulare Co.: Whitaker Forest, E.EshomCrk.Drainage, nr. tree#142, 36.7062°N, 118.9319°W, 1650masl, MT, 3.vi–16.vii.2010 P.H. Kerr CSCA10L174” [CSCA]; 8 ♂♂, 3 ♀, “USA: CA: Tulare Co: Whitaker’s Forest, Ridge S. of Eshom Crk., 1620masl, 36.7011°N, 118.9363°W, MT, 3.vi–16.vii.2010 P.H. Kerr CSCA10L175” [CSCA]; 4 ♂♂, 2 ♀, “USA: CA: Tulare Co.: Whitaker Forest, E.EshomCrk.Drainage, nr. tree#142, 36.7062°N, -118.9319°W, 1650masl, YPT, 3.vi–16.vii.2010 P.H. Kerr CSCA10L258” [CSCA]; 1 ♂, “USA: CA: Glenn Co., Atchison Campsite pine forest, ex: Malaise, elev. 1310m, 20–24.v.2012, colls: K. Will, K. Yao, N. Grady-Grot, 39°45'00"N, 122°55'33"W, CAL2012.v.23.5” [EMEC].

#### Diagnosis.

This species may be distinguished by the characters of the genus and by the male genitalia; particularly the gonostylus which has two apical lobes, the ventral one being distinctly sclerotized and darkened.

#### Description.

Male. Body length (n=4): 4.6–6.6, 5.7 [4.6] mm. Wing length: 5.3–5.9, 5.6 [5.9] mm (n=4).

**Figure 1.**
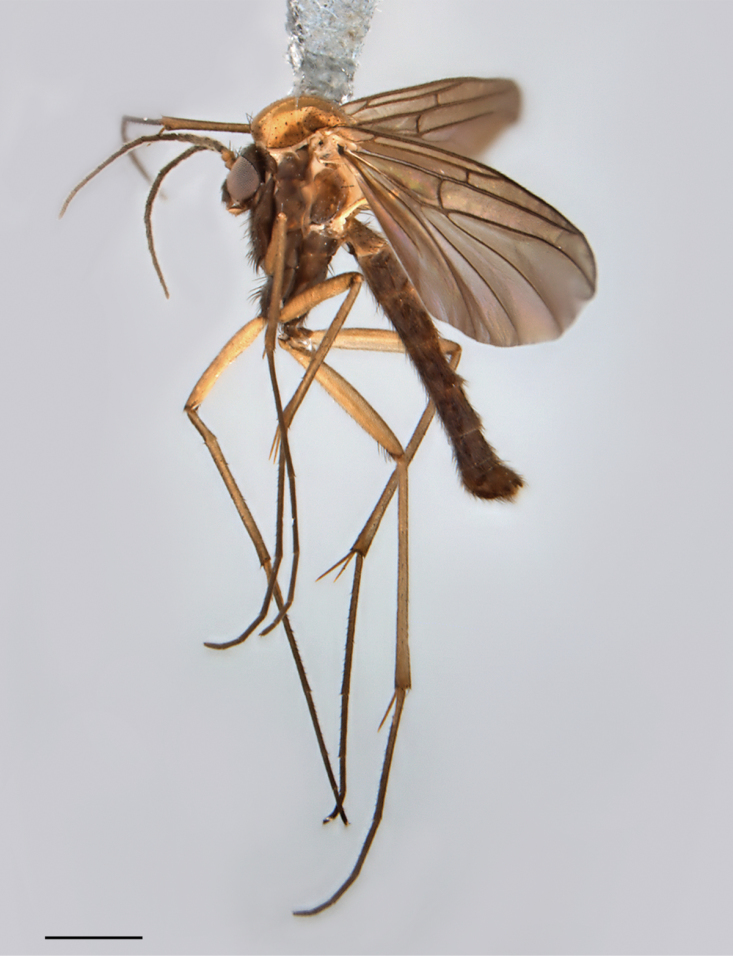
*Spritella sequoiaphila* sp. n., habitus [holotype male, # 10F761]. Scale bar = 1 mm.

**Figure 2. F2:**
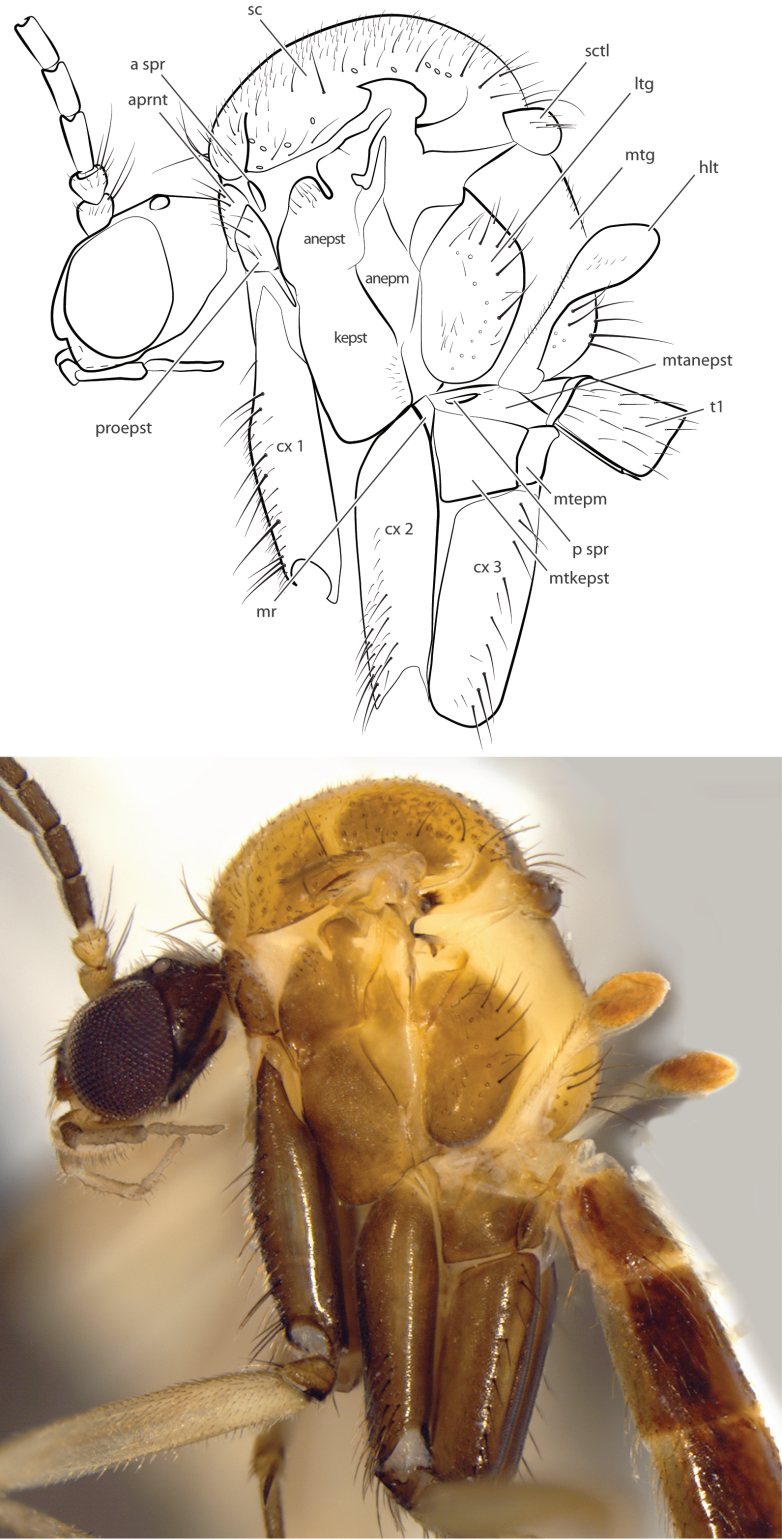
*Spritella sequoiaphila* sp. n., thorax, lateral view [paratype male, #11G040]. Abbreviations: **anepm** anepimeron **anepst** anepisternum **aprnt** antepronotum **a spr** anterior spiracle **cx** coxa **hlt** halter **kepst** katepisternum **ltg** laterotergite **mr** meron mtg mediotergite **mtanepst** anepisternum **mtepm** metepimeron **mtkepst** metakatepisternum **p spr** posterior spiracle **patg** paratergite **proepm** proepimeron **proepst** proepisternum **sc** scutum **sctl** scutellum.

*Coloration* (Figs 1, 2). Head brown; face and clypeus brown; palpomeres light brown to brown. Antennal scape and pedicel yellowish light brown, base of first flagellomere yellowish light brown, otherwise brown; all remaining flagellomeres brown. Thorax variously yellow, yellowish to orangish brown to brown; scutum yellowish light brown dorsally, with faint brown band postero-medially, orangish brown to brown laterally; scutellum light brown to brown; antepronotum, proepisternum, anepisternum, and katepisternum brown; anepimeron light brown; laterotergite and anepisternum brown; metepimeron and metakatepisternum brown to dark brown; halteres yellowish light brown; mediotergite yellowish light brown. Wing membrane lightly brown infuscated. Coxae dark brown; femora yellowish light brown; tibiae slightly darker yellowish light brown; tarsi brown. Abdominal segments light brown to brown, lighter in color near anterior margin on segments 1–6. Terminalia brown.

*Head*. Ocelli slightly raised; middle ocellus smaller than (approx. 0.5× size of) lateral ocelli. Face with golden brown setae laterally, mostly bare medially. Antenna length 2.7–3.0, 2.9 [2.7] mm (n=4); about 2× length of thorax, shorter than abdomen. Palpus with four visible palpomeres, slightly longer than width of head (anterior view); length of palpomeres 1 and 2 nearly subequal (palpomere 2 longer); palpomere 3 approx. 5× longer than wide; palpomere 4 approx. 10× longer than wide, subequal to or shorter than combined length of palpomeres 1–3.

**Figure 3. F3:**
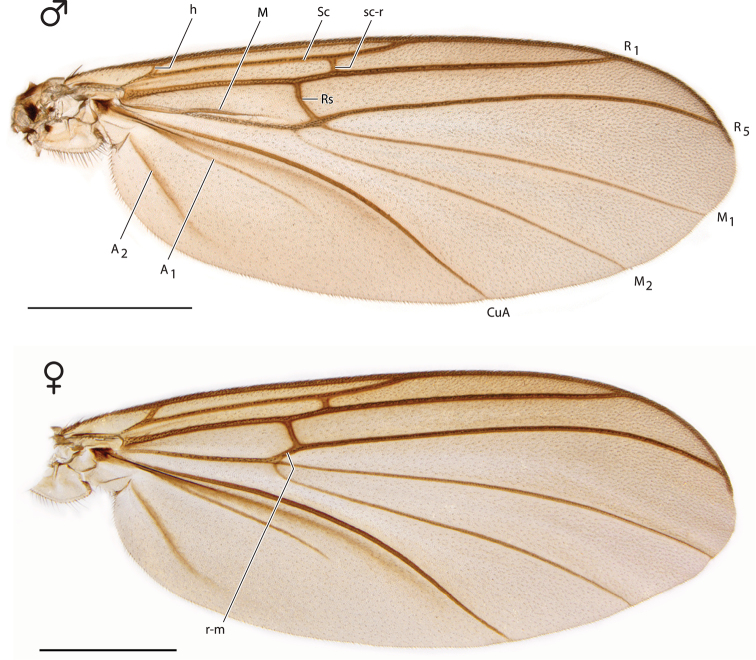
*Spritella sequoiaphila* sp. n., right wings [paratype male, #10F296; paratype female #14P342]. Scale bar = 1 mm.

**Figure 4. F4:**
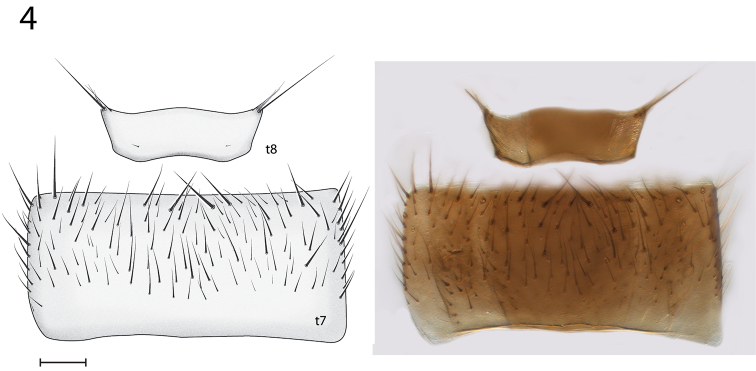
*Spritella sequoiaphila* sp. n., male abdominal tergites 7–8 [holotype, # 10F761]. Sternite 7 slightly visible below. **t** tergite. Scale bar = 0.1 mm.

**Figure 5. F5:**
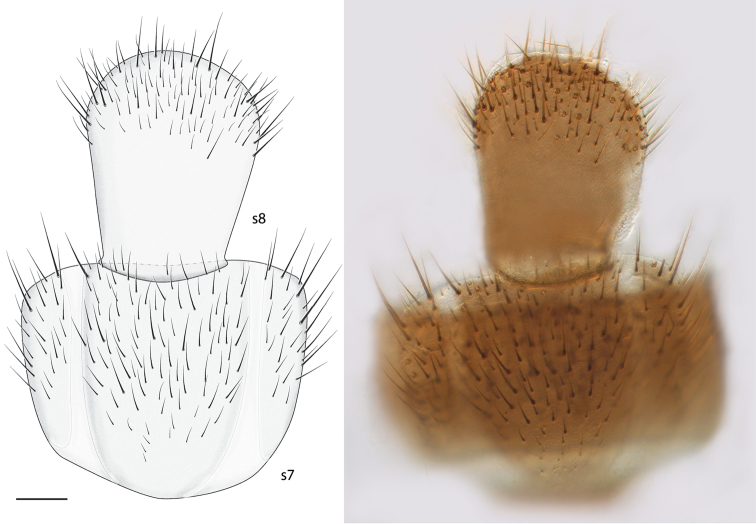
*Spritella sequoiaphila* sp. n., abdominal sternites 7–8 [holotype male, # 10F761] (looking through t7). **s** sternite. Scale bar = 0.1 mm.

*Thorax and Abdomen*. Wing as Fig. 3; Rs distinctly elongate; vestigial M vein present within discal cell; M_1_ arises from R_4+5_ at origin of Rs so that r-m not present; M_2_ weak at base. Tergite 8 reduced (Fig. 4), with setae on posterolateral margin, and a pair of setulae sub dorsally; sternite 8 (Fig. 5) elongate, setose on posterior half, approximately half width of sternite 7.

**Figures 6–7. F6:**
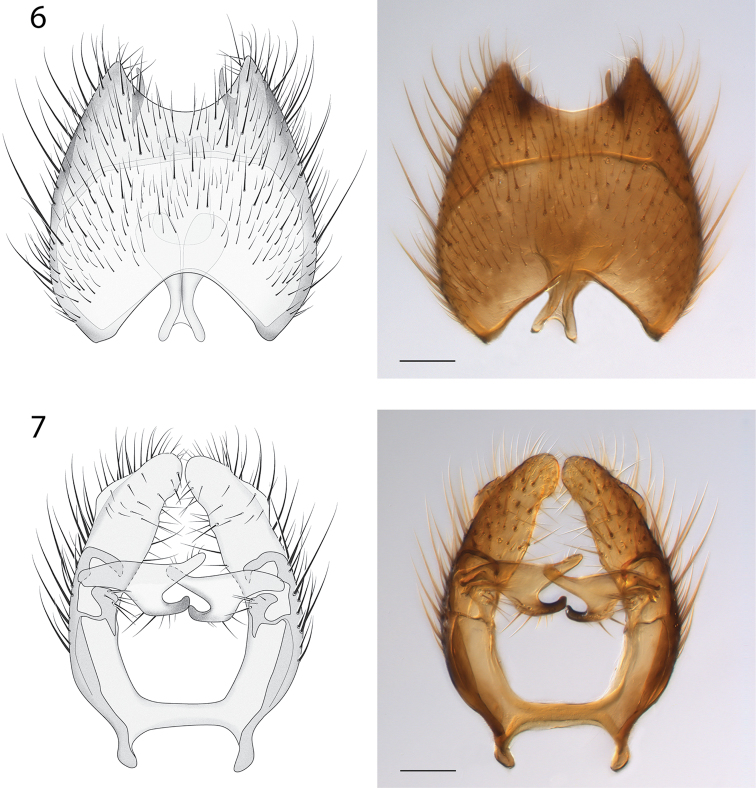
*Spritella sequoiaphila* sp. n., illustrations and photomicrographs of structures of the male genitalia, dorsal view [holotype male, # 10F761]. **6** epandrium **7** gonocoxites. Images same scale, scale bar = 0.1 mm.

**Figures 8–9. F7:**
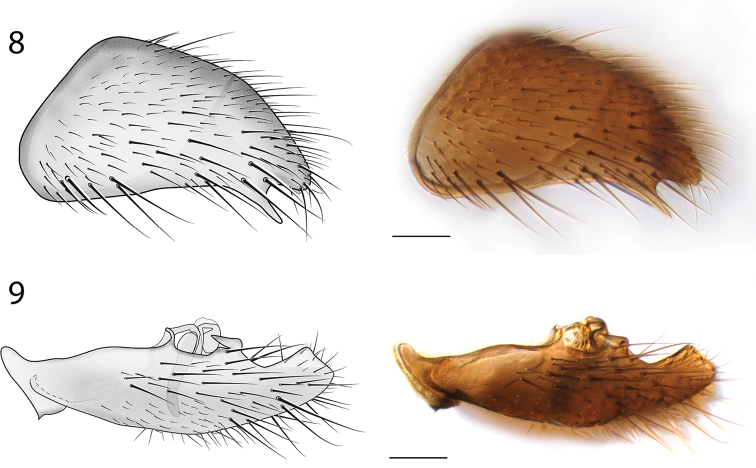
*Spritella sequoiaphila* sp. n., structures of the male genitalia, lateral view [holotype male, # 10F761]. **8** epandrium **9** gonocoxites. Scale bar = 0.1 mm.

*Male Genitalia* (Figs 6–9). Epandrium deeply emarginate both anteriorly and posteriorly, with pair of submedial apical fins (Fig. 6) and pair of short lateral processes (Fig. 8). Gonostyli with pair of setose apical lobes, the ventral one with dark sclerotization.

Female. Body length: 4.4–5.5, 5.1 mm (n=4). Wing length: 4.8–5.3, 5.1 mm (n=4). Antenna length 1.9–2.2, 2.1 mm (n=4).

*Coloration*. Similar to male.

*Thorax*. Wing as Fig. 3; Rs not as long as in male; vestigial M vein within discal cell absent; r-m present; M_2_ arises at or near base of M_1_.

**Figures 10–11. F8:**
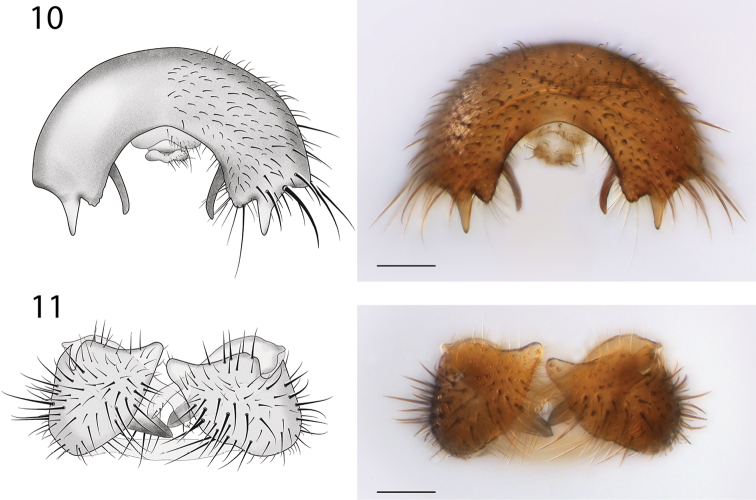
*Spritella sequoiaphila* sp. n., structures of the male genitalia, posterior view [holotype male, #10F761]. **10** epandrium **11** gonocoxites. Scale bar = 0.1 mm.

*Female Genitalia* (Fig. 10). Tergite 8 narrow, expanded laterally; first cerci fused, approx. 2× longer than wide; second cerci ovoid; hypoproct elongate, subtending most of cercus 1; sternite 8 with deep medial cleft reaching anterior margin.

**Figure 12–13.**
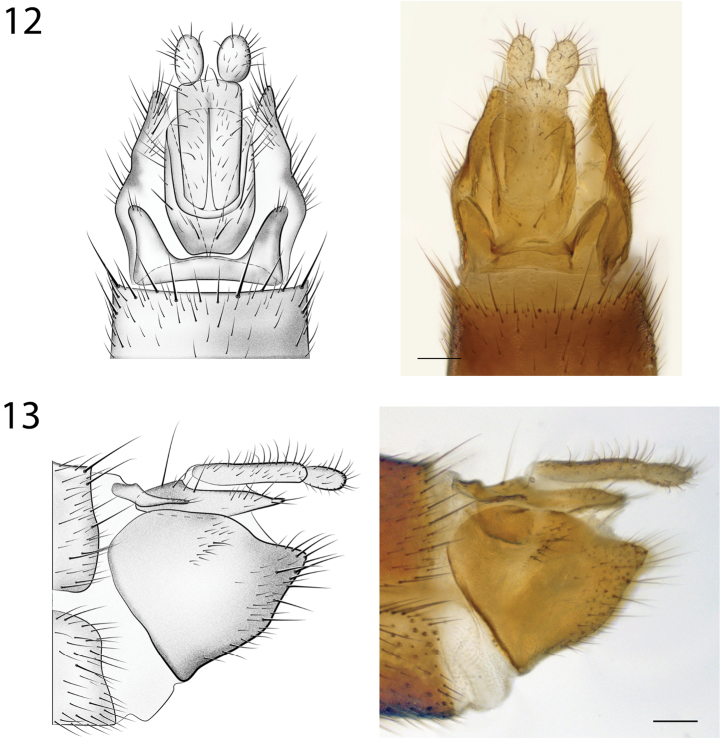
**12** *Spritella sequoiaphila* sp. n., structures of the female genitalia, dorsal view [# 14P342]. Scale bar = 0.1 mm **13** *Spritella sequoiaphila* sp. n., structures of the female genitalia, lateral view [# 14P342]. Scale bar = 0.1 mm

#### Etymology.

The species epithet is an adjective, referring to its affiliation with giant sequoia groves (“sequoia-loving”).

## Phylogenetic analysis

The phylogenetic analysis was carried out by adding *Spritella sequoiaphila* gen. et sp. n. to the scored character matrix created by Borkent and Wheeler (2013), with minor modifications (Suppl. material 1). The following changes were made to avoid problems of non-independence: Character # 43, wing macrotrichia orientation (decumbent or reflexed), was changed to “?” for taxa whose wing macrotrichia was scored as absent (41: 1). For taxa whose ventral surface of subcostal vein was scored as bare (54: 0), character # 55 was not applicable and changed to “?” since the presence or absence of setae on the ventral base of subcostal vein was not free to vary (it was already scored as bare). The same type of non-independence was found in character # 57 which treats the dorsal side; the subcostal vein (bare or setose), was changed to “?” for taxa that had already been scored as bare in the previous character (56: 0). Character # 68, position of anterior fork origin, was changed to “?” for taxa whose anterior fork (M1 + M2) was scored as absent (67: 1). For taxa whose posterior fork was scored as absent (73: 1), characters involving aspects of the posterior fork (74–76, 79) were changed to “?”. Character #30, anapleural suture (single, double, or absent), was re-scored as “single” (30: 0) for *Acomoptera plexipus* (Garrett) and *Acomoptera vockerothi* Kerr since this character state could be verified with specimens in the CSCA collection.

All characters were scored for *Spritella sequoiaphila* except the following: 47, humeral vein (oblique or curved); 84, arrangement of vestiture of tibia (irregular, apical portion with parallel lines, or all in parallel lines); and 87, vestiture arrangement on tarsomeres (irregular or in parallel lines). Due to problems of interpretation and reproducibility, these characters were scored as “?” in the final matrix; I could not score these characters confidently for any taxa, including those used in the original Borkent and Wheeler (2013) matrix. For wing characters, the female (Fig. 3) was used to facilitate homology recognition.

The modified matrix was analyzed using parsimony; 1000 heuristic search replicates and 500 bootstrap replicates were performed using PAUP* 4.0b10 (Swofford 2001), with random-taxon-addition, tree bisection reconnection (TBR) branch swapping, steepest decent and ‘MulTrees’ options in effect. All characters were treated unordered and assigned equal weights. Mesquite (Maddison and Maddison 2011) was used to analyze character change and support in the phylogenetic tree.

## Phylogenetic results and discussion

**Figure 14.**
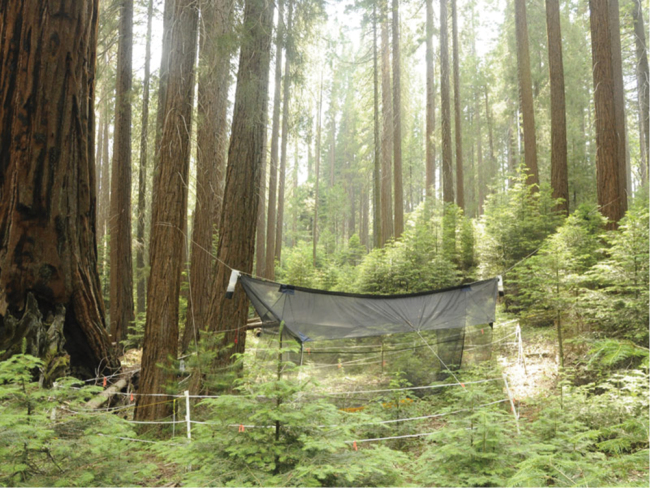
Locality of *Spritella sequoiaphila* sp. n., showing 6-meter Malaise trap; Whitaker Forest (UC Berkeley Center for Forestry), Tulare Co. [CSCA10L258].

Parsimony heuristic searches found 61 most parsimonious trees (Fig. 14). The strict consensus of these trees differs from the results provided by Borkent and Wheeler (2013), although the underlying data matrix is largely the same. Bootstrap values above 50% support the monophyly of most genera while support for intergeneric relationships is largely lacking. A monophyletic Sciophilinae includes *Syntemna hungarica* Lundström and its sister lineage, although excludes species of *Aneura* Marshall, a genus normally thought to be included in the subfamily. Inside this group are two major lineages that contain the remaining taxa, sister to *Taxicnemis marshalli* Matile. Using implied weighting, Borkent and Wheeler (2013) may have found a similar tree, with *Syntemna* Winnertz as the sister to the rest of the Sciophilinae and *Phthinia* Winnertz and *Polylepta* Winnertz united as sister taxa.

The concept of the “*Azana* group” (Matile 1998) is recovered with the inclusion of *Morganiella fusca* Tonnoir and *Paramorganiella adventurosa* Tonnoir. This result is consistent with the composition of this group as suggested by Amorim and Oliveira (2008), although both *Morganiella* Tonnoir & Edwards and *Paramorganiella* Tonnoir have a complete posterior fork (character 73:0) unlike the other members of this group. *Sciophila* Meigen, which forms a trichotomy and may or may not be included in this group, also has a posterior fork that is complete (or interrupted basally; as in *Sciophila interrupta*, *Sciophila cincticornis*, and *Sciophila fractinervis*). Within this clade, the new genus is included.

*Spritella sequoiaphila* is recovered sister to *Afrocnemia whitfieldae* Matile. The two genera are united by the ocellar arrangement being linear (6: 2) and the first flagellomere being slightly offset (20: 1). Along with most species of *Acnemia*, *Afrocnemia whitfieldae* and *Spritella sequoiaphila* have a distinctively short anterior wing vein fork. This character is difficult to score discretely for phylogenetic analysis (e.g., at what point is it “short” or “long”?) and was not scored by Borkent and Wheeler (2013) but appears to have phylogenetic signal that should be considered for future analyses.

*Acnemia* is recovered as a paraphyletic group, which is not especially surprising, since members of this genus can vary widely and in a way that suggests that this group is in need of systematic revision. Borkent and Wheeler (2013) did find that *Acnemia* are united by a unique synapomorphy, however, in that the gonostylar lobe bears one to three thin processes. These processes are lacking in *Spritella sequoiaphila*.

**Figure 15.**
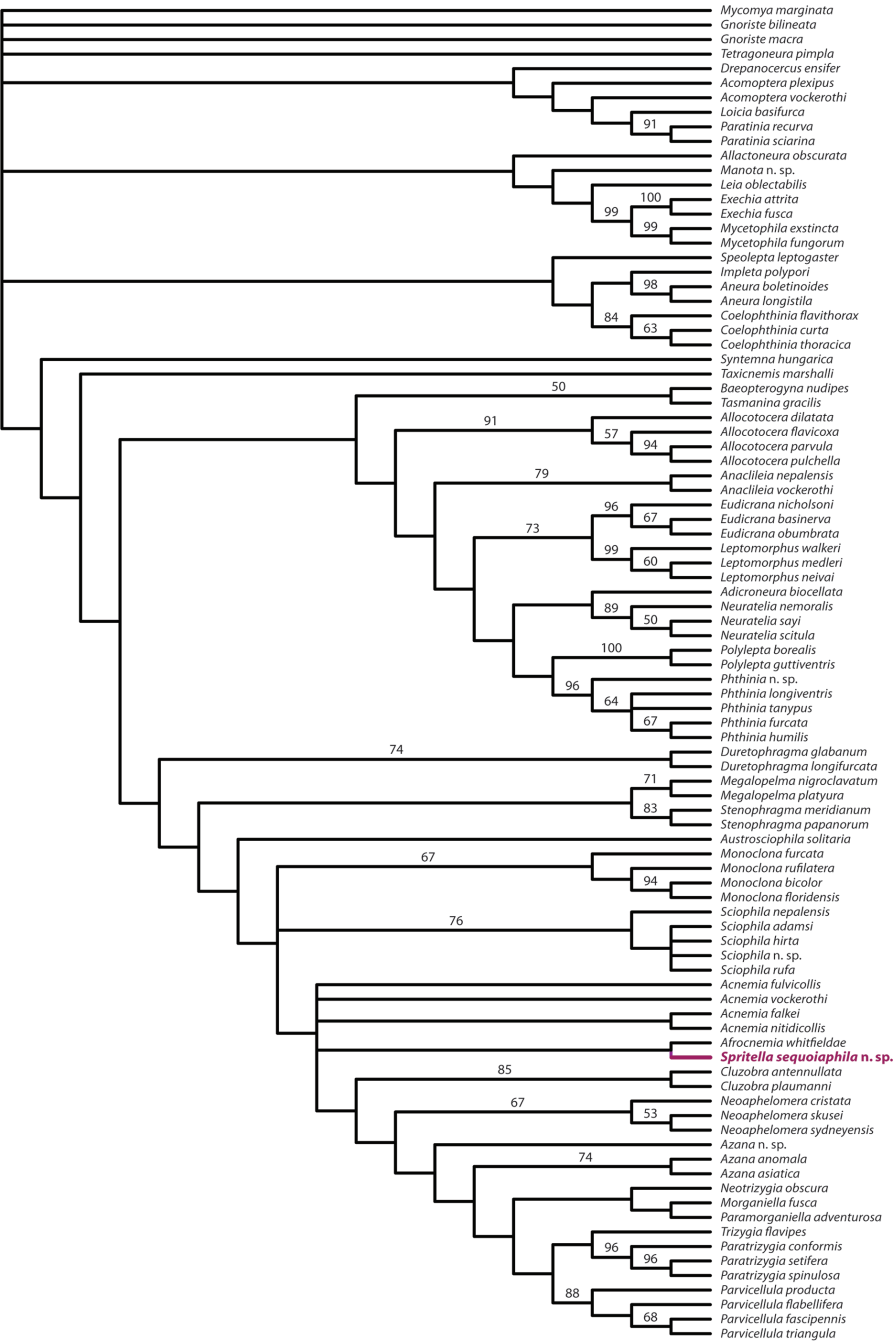
Strict consensus tree of 61 equally parsimonious trees from re-analysis of Borkent and Wheeler (2013), with the addition of *Spritella sequoiaphila* sp. n. Bootstrap values provided above branches where support ≥ 50% (500 reps).

## Supplementary Material

XML Treatment for Spritella

XML Treatment for Spritella sequoiaphila

## Supplementary material

Supplementary material 1 Data matrix in nexus format (modified from Borkent and Wheeler 2013) doi: 10.3897/zookeys.437.7932.app Data type: NEXUS file with DNA data Explanation note: Data matrix in nexus format (modified from Borkent and Wheeler 2013). This dataset is made available under the Open Database License (http://opendatacommons.org/licenses/odbl/1.0/). The Open Database License (ODbL) is a license agreement intended to allow users to freely share, modify, and use this Dataset while maintaining this same freedom for others, provided that the original source and author(s) are credited.Peter Kerr
